# Exploring the Potential of Isalo Scorpion Cytotoxic Peptide in Enhancing Gill Barrier Function and Immunity in Grass Carp (*Ctenopharyngodon idella*) Infected with *Flavobacterium columnare*

**DOI:** 10.1155/2024/8059770

**Published:** 2024-07-24

**Authors:** Qiu-Yan Chen, Qi-Yu Hu, Wei-Dan Jiang, Pei Wu, Yang Liu, Hong-Mei Ren, Xiao-Wan Jin, Lin Feng, Xiao-Qiu Zhou

**Affiliations:** ^1^ The Animal Nutrition Institute Sichuan Agricultural University, Chengdu 611130, China; ^2^ University Key Laboratory of Sichuan Province of Fish Nutrition and Safety Production Sichuan Agricultural University, Chengdu 611130, China; ^3^ Key Laboratory of Animal Disease-Resistant Nutrition Ministry of Education, Chengdu 611130, China; ^4^ Key Laboratory of Animal Disease-Resistance Nutrition Ministry of Education Ministry of Agriculture and Rural Affairs Key Laboratory of Sichuan Province, Sichuan 611130, China

## Abstract

The objective of this research was to investigate how dietary antimicrobial peptides (AMP), namely, Isalo scorpion cytotoxic peptide (IsCT), affect the gill physical barrier function and immune function of grass carp challenged with *Flavobacterium columnare* (*F. columnare*). Five hundred forty grass carp were randomly allocated to six groups and fed to varying levels of IsCT in the diet (0, 0.6, 1.2, 1.8, 2.4, and 3.0 mg/kg diet) for a duration of 60 days. Afterward, the grass carps faced a challenge from *F. columnare*. The results revealed that the use of optimal IsCT dramatically mitigated gill damage in grass carp that were infected with *F. columnare*. Additionally, IsCT exhibited a notable enhancement in gill antioxidant capabilities, as evidenced by a significant reduction in ROS, MDA, and PC levels, an elevation in antioxidant enzyme activities, and an upregulation of antioxidant-related genes and *Nrf2* mRNA levels. Conversely, the expression of *Keap1a* and *Keap1b* mRNA was decreased. Besides, IsCT exhibited its capability to inhibit apoptosis via downregulating the mRNA levels of *caspase-2*, *caspase-3*, *caspase-7*, *caspase-8*, *caspase-9*, *Apaf1*, *Fasl*, *Bax*, and *JNK* while concurrently increasing the mRNA levels of *Bcl-2*, *Mcl-1*, and *IAP* in fish gills. Additionally, IsCT promoted the integrity of tight junction barrier by increasing the gene expression of *claudin-b*, *claudin-c*, *claudin-3c*, *ZO-1*, *ZO-2b*, *occludin*, and *JAM* while suppressing MLCK signaling. Additionally, optimal dietary IsCT improved antibacterial ability, as evidenced by heightened LZ, ACP activities, and elevated levels of C3, C4, and IgM. Additionally, there was an upregulation in *β-defensin-1*, *LEAP-2A*, *LEAP-2B*, *hepcidin*, and *mucin-2* mRNA expression in the gills. Simultaneously, the inclusion of optimal dietary IsCT in the diet resulted in improved gill immunity barriers through the reduction of proinflammatory cytokine mRNA levels and the increase in the expression of anti-inflammatory cytokine mRNA levels. This was partly facilitated by the I*κ*Ba/NF-*κ*B p65 signaling pathway and TOR/S6K1 signaling pathways in the gills of grass carp. Therefore, supplementing the diet with IsCT has potential advantages in enhancing gill health by improved physical barriers and immunity in grass carp. Based on LZ activity and against lipid peroxidation, optimum IsCT concentrations in on-growing grass carp (136.88 ± 0.72 g) were found to be 1.68 and 1.54 mg/kg diet, respectively.

## 1. Introduction

The rapid advancement of aquaculture has strengthened fish culture to meet the growing demand for protein-rich food [1]. Nevertheless, intensive aquaculture frequently results in pathogenic microbial infections in fish, subsequently causing outbreaks of disease and significant negative economic consequences [2]. As is well-known, fish live in intimate contact with the environment; the gill serves as the first-line of encounter with pathogens and exerts a crucial role in fish mucosal immune defense, which relies on the gill's structural integrity [3, 4]. Hence, the protection of gill well-being, encompassing various aspects like physical barrier integrity and immune function, holds paramount significance in fish and serves as an optimal approach to safeguarding aquatic creatures against diseases. In recent years, one of the most promising methods has been to strengthen fish defense mechanisms through dietary nutrition stimulus.

Antimicrobial peptides (AMPs) are abundantly found in organisms and play a pivotal role in modulating innate immune functions against pathogens; due to their natural antimicrobial properties and relative safety, they have received attention with regard to fish [2, 5]. The antimicrobial peptide employed in this investigation, Isalo scorpion cytotoxic peptide (IsCT), was characterized as the shortest *α*-helical antimicrobial peptide possessing antimicrobial properties [6]. A prior investigation conducted by our research team showed that the inclusion of IsCT in diet enhances growth performance, attenuates intestinal histopathological lesions, and improves intestinal immune function in grass carp [7]. However, there have been no prior research investigating the potential effects of IsCT on the gill health in fish. According to Alesci et al. [8], the gill mast cells of teleost giant mudskipper (*Periophthalmodon schlosseri*) exhibited high level of expression for the antibacterial peptide Piscidin1. The study reveals that Cecropin P1 antimicrobial peptide has the ability to enhance the immune response in the gill cell line of rainbow trout (*Oncorhynchus mykiss*) [9]. Hence, it is logical to hypothesize that there could be a correlation between IsCT and the well-being of fish gills, which is the subject of examination in this research.

Physical barriers are widely recognized as the primary defense mechanism against external pathogens, playing a crucial role in keeping the overall health of fish gills. The integrity of the gills' structure is closely linked to the cellular integrities and intercellular tight junctions (TJs) complexes [2, 10]. It is worth noting that apoptosis and oxidative damage can impaired the epithelial cells of the gills. Earlier research has suggested that oxidative damage, apoptosis, and intercellular TJs could be mediated by nuclear factor erythroid 2-related factor 2 (Nrf2), c-Jun N-terminal protein kinase (JNK), and myosin light chain kinase (MLCK) pathways, respectively [11, 12]. A study found that feeding yellow croaker *Larimichthys crocea* with the antimicrobial peptide APSH-07 increased antioxidant enzyme activity in the liver [13]. Additionally, the human antimicrobial peptide LL-37 was found to inhibit caspase 3 activity in endothelial cells [14] and inhibit the spontaneous apoptosis of neutrophils [15]. By inhibiting the activation of MLCK, the cathelicidin peptide C-BF enhanced LPS-induced reduction in *occludin* and *ZO-1* mRNA of mouse intestinal [16]. Therefore, there may be a correlation between IsCT and physical barrier of fish gill, which deserves further investigation.

In gills, the balance between immune responses to waterborne pathogens and suppressing inflammation is critical for effective overall fish health [10]. Research has indicated a strong correlation between gill immune function and various immunological parameters such as inflammatory cytokines, lysozyme, and antibacterial compounds [17]. Nuclear factor *κ*B (NF-*κ*B) plays an importance role as a transcription factor in controlling the inflammatory response. NF-*κ*B and the target of rapamycin (TOR) signaling pathways facilitate the transcriptional mediation of inflammatory cytokines in fish, ultimately leading to an immune response [18, 19]. Gills and intestines play crucial roles as immune-relevant tissues in fish. The immune capacity of the intestines is a determining factor in the absorption and overall health of fish. Furthermore, the immune capacity of fish gills, being the primary organ for respiration and excretion, significantly influences the growth and survival of the fish. Recent research conducted on zebrafish has demonstrated that dietary AMPs can enhance the expression of immune-related genes, including *TNF*-*α*, *lysozyme*, *IL-1β*, and *IL*-*8* mRNA, within the intestine region [20]. Furthermore, in the presence of a challenge posed by *Aeromonas hydrophila*, antimicrobial peptides have been discovered to enhance the levels of gene expression for *IL-10* and *TGF-β* while reducing the levels of gene expression for *TNF-α*, *IL-1β*, and *IL-8*. This mechanism may enhance the grass carp's intestinal immune responses against pathogen invasion [21]. In mice, the NF-*κ*B signaling pathway was inhibited by cathelicidin-BF, an antimicrobial peptide, leading to the suppression of TNF-*α* secretion [16]. However, there is a lack of previous research examining the potential impacts of IsCT on the health of fish gills. Thus, it is of great importance and necessity to determine whether IsCT correlates with gill immunity.

Grass carp, a commonly cultivated freshwater fish known for its well growth and high yield, serves as the focal species for this investigation. In spite of that, grass carp, as a kind of low vertebrate animal, is easy to be infected by pathogens in the process of culture and then cause diseases such as rotten gills, enteritis. Microorganisms, such as *F. columnare*, commonly exist as pathogens in freshwater environments and serve as the causative agent of columnaris disease, which seriously affects the health of fish and causes damage to various tissues, including the skin, fins, and gill mucosal tissues. As a part of larger studies, this research aims to comprehensively evaluate the effects of IsCT on the structural and immune barriers following gill infection with *F. columnare* in grass carp, so as to provide theoretical basis and important guidance for healthy aquaculture.

## 2. Materials and Methods

### 2.1. The Experimental Conditions, Diets, and Grass Carp Prepared Accordingly

The basal diet's formulation and relative chemical composition are shown in Table S1, which was previously published by Hu et al. [7]. The IsCT, with a molecular weight of 1.5 kDa, was sourced of Sun Smile Biotechnology Co., Ltd. (Shenzhen, China). The basal diet was gradually augmented with incremental levels of IsCT (0, 0.6, 1.2, 1.8, 2.4, and 3.0 mg/kg diet) to prepare six experimental diets.

The healthy grass carps were obtained from fisheries (Sichuan) and were fed a basal diet for 2 weeks. Then, 540 grass carps with a mean initial weight of 136.88 ± 0.36 g were randomly selected in a random manner and subsequently allocated among 18 cages, with each cage accommodating 30 fish. Each treatment consisted of three cages. Artificial feeding was given to the grass carps four times daily to apparent satiation, while a disk with a diameter of 100 cm was placed at the bottom of each cage to gather any leftover feed. This feeding experiment continued for a duration of 60 days. Dissolved oxygen, temperature, and pH of the monitored water were kept at 6.0 mg/L, 26.5–30.5°C, and 7.2–7.8, respectively.

### 2.2. Grass Carps Challenge with *F. columnare* and Gill Sample Collection

The challenge trials began after 60 days of the feeding experiment. The preparation of the pathogenic strain followed the methodology outlined in our prior studies [22]. After conducting the feeding trials, fish with the same weights from each treatment group (3 small cages/group, *N* = 5 fish/small cage) were randomly chosen and immersed with *F. columnare* (1 × 10^8^ CFU mL^−1^) for a duration of 3 hr in a challenge cage. After that, the grass carps were returned to their original test cage for 3 days. The challenges were conducted under the same conditions as those mentioned above. After the infection trial, for each replicate, five fish were randomly chosen and subjected to anesthesia using a 50 mg/L benzocaine bath. To assess the severity of gill damage, a grading system similar to that employed in previous research was utilized [23]. Then, the fish were carefully dissected to isolate gill samples from each individual fish, a part of gill tissue was embedded in 10% neutral buffer formalin for histology analysis, and another part was pooled in cryopreservation tubes and immediately stored in −80°C before further processing.

### 2.3. Evaluation of Biochemical Parameters and DNA Fragmentation

The gill was homogenized by adding it to phosphate buffer (10 : 100, w/v) and after that at a condition of 3,500 g, 4°C centrifuge for 10 min. During the subsequent stage, the transparent liquids were packaged separately to measure the activity and composition of the enzymes. According to the manufacturer's instructions, kits (Jiancheng, Nanjing, China) were used to measure the activities of ASA, AHR, CAT, lysozyme, GPx, GST, GR, SOD, and contents of GSH, T-AOC, complement 3(C3), C4, MDA, PC, IgM, and ROS.

The method described by Wang et al. [22] was utilized to assess the fragmented DNA of the grass carp's gill. The Apoptosic DNA Ladder Extraction Kit was utilized, and electrophoresis was conducted on a 2% agarose gel at 40 V for 1.5 hr. Syngene's Gene Genius Bio-Imaging system was used to examine and capture images of the gel (Frederick, MD, USA).

### 2.4. Analysis of Gene Expression

Total RNA from the grass carp gills was extracted using the TaKaRa RNA Trizol kit (Dalian, China) on the basis of instructions provided by the manufacturer. The NanoPhotometer reading at 260/280 nm was used to evaluate the integrity and purity of the RNA (with a 260/280 ratio of 1.8–2.0 for purified RNA), and the RNA quality was confirmed by 1% agarose gel electrophoresis. Afterward, PrimeScript™ RT reagent Kit (Takara, Dalian, China) was used to synthesize cDNA synthesis from the total RNA as the starting material. The detection of gene expressions was performed using real-time qPCR, with the quantitative primers for reference genes (*β*-actin) and target genes listed in Table S2. The relative expression contents of genes after determining the primers amplification efficiency amount were analyzed by 2 ^−*ΔΔ*CT^ following the method described via Livak [24].

### 2.5. Western Blotting Analysis

The glass tissue grinder was used to homogenize the gills, and the Protein Quantification Analysis Kit (Beyotime Biotechnology Inc., China) was used to measure the content of homogenization protein. All gill tissue protein samples were diluted to the same concentrations. Separate the gill protein samples with SDS-PAGE (10%), and then shift them to a PVDF membrane with a pore size of 0.45 *μ*m, for western blotting. Then the membrane was incubated with primary antibody for 12 hr at 4°C. Then, the membranes were rinsed four times for 5 min each, followed by a 90 min incubation with a secondary antibody. After incubation, protein signals were visualized, and the western bands were quantified with the help of ImageJ software.

### 2.6. Calculations and Statistical Methods

In order to analyze the data, SPSS 26.0 was used, which were then presented as mean ± standard deviation (SD). The significance of the observed changes was evaluated through the utilization of one-way analysis of variance (ANOVA). Further verification was performed using Duncan's multiple range test. At the same time, the linear and quadratic effects of varying doses of IsCT were analyzed by orthogonal polynomial contrasts. The distinction between the various experimental groups was evaluated at a significance level of *P* < 0.05. All visual representations were generated using OriginPro 2017C.

## 3. Results

### 3.1. Rot Morbidity and Histopathology in Grass Carp Gill

**Figure 1:**
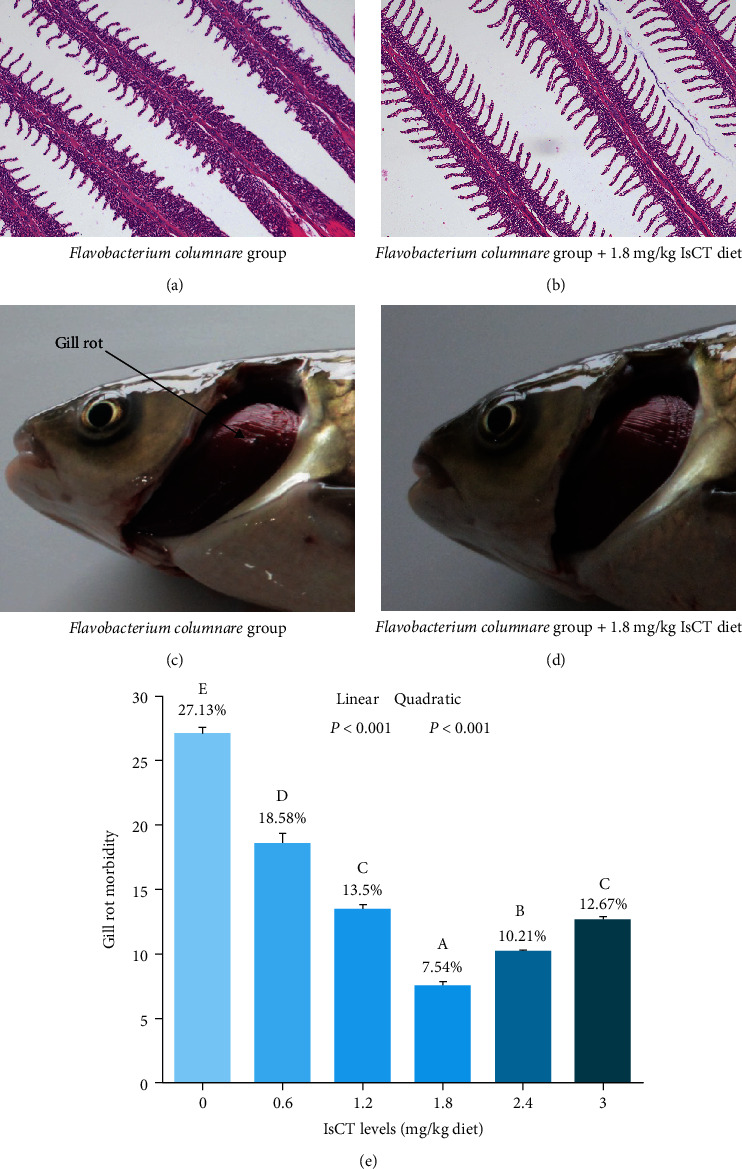
Gill histological sections (a and b), gill rot symptoms (c and d), and gill rot morbidity (e) of grass carp. Values are means ± SD, *n* = 6 (six fish in each group). (a and c) *Flavobacterium columnare* group. (b and d) *Flavobacterium columnare* +1.8 mg/kg IsCT diet. Different letters indicate significant differences (*P* < 0.05). *P* values indicate a significant linear or quadratic dose–response relationship (*P* < 0.05).

Figure 1 shows the results of IsCT on the gill rot morbidity and histopathology of grass carp. The grass carp gill rot morbidity was significantly reduced in a linear or quadratic manner when fed with IsCT, and the group of grass carp fed 1.8 mg/kg IsCT showed the most effective protection against gill rot (*P* < 0.05). Meanwhile, the histology results exhibited that the proper levels of IsCT treatment markedly relieved the severe capillary hematoma and gill damage following *F. columnare* infection.

### 3.2. Antioxidant Parameters of Grass Carp Gill

**Figure 2:**
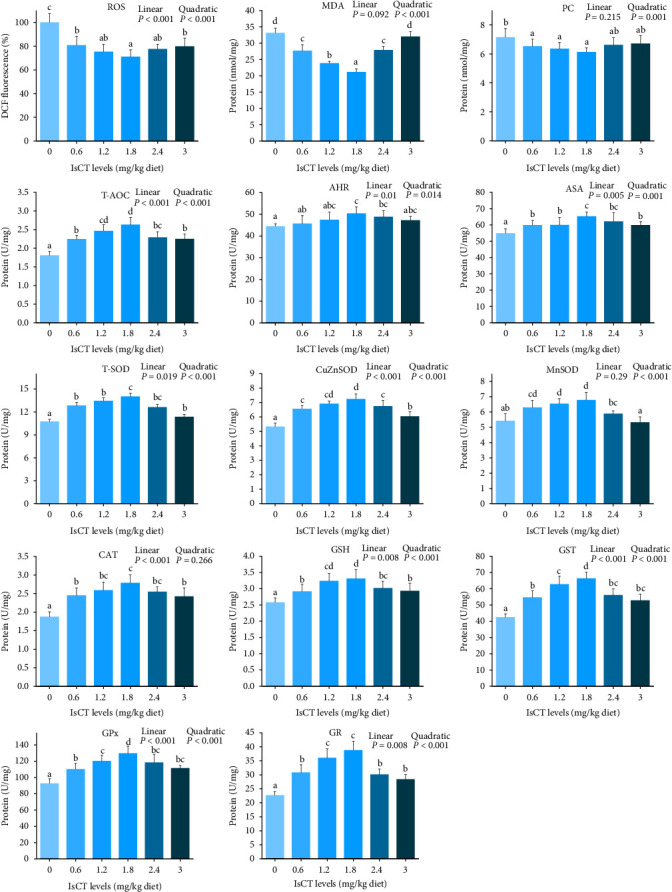
The impact of IsCT in the diet on grass carp gill antioxidant status. Values are means ± SD, *n* = 6 (six fish in each group). Different letters indicate significant differences (*P* < 0.05). *P* values indicate a significant linear or quadratic dose–response relationship (*P* < 0.05).

Figure 2 shows how IsCT affects antioxidant parameters of grass carp gills. The IsCT treatment resulted in a notable decrease in ROS production across all groups (*P* < 0.05), with a dramatically linear or quadratic reduction effect (*P* < 0.001). In the 0.6–2.4 mg/kg group, MDA content was remarkably diminished (quadratic, *P* < 0.05). The addition of 0.6–1.8 mg/kg led to a decrease in PC content (quadratic, *P* < 0.05), with the most significant impact observed in the group receiving 1.8 mg/kg. The activities of T-AOC, AHR, T-SOD, ASA, GR, GST, CAT, CuZnSOD, and GPx and the content of GSH initially rose and subsequently declined with the rise in IsCT concentration in the diet from 0 to 3.0 mg/kg (linear or quadratic, *P* < 0.05), and these parameters reached the maximum activities or contents in 1.8 mg/kg addition group (*P* < 0.05). The MnSOD activities of fish fed IsCT 0.6–2.4 mg/kg concentrations were found to be higher than those of any group (*P* < 0.05).

**Figure 3:**
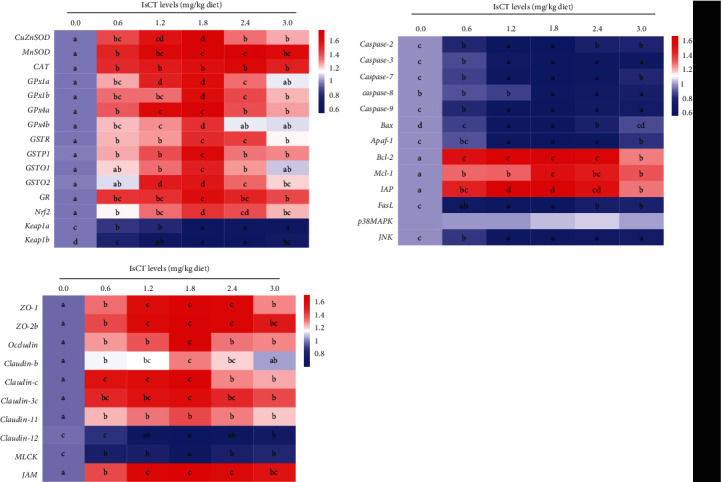
Heat-map displays alterations in the expression of genes and signaling factors related to antioxidant, apoptosis, and tight junction in grass carp gills. Different letters indicate significant differences (*P* < 0.05).

According to Figure 3, the upregulation of antioxidant-related genes, such as *CuZnSOD*, *GPx1a*, *GPx4a*, *GPx1b*, *GPx4b*, *GSTR*, *GSTP1*, *GSTO2*, *MnSOD*, and *GSTO1*, and *GR* expressions in grass carp gills reaches the highest level at 1.8 mg/kg. However, after that, there was a decreasing trend in their expressions (*P* < 0.05). Additionally, the addition of various concentrations of IsCT to the diet resulted in a substantial increase in *CAT* mRNA expression in the gills of grass carp (*P* < 0.05).

**Figure 4:**
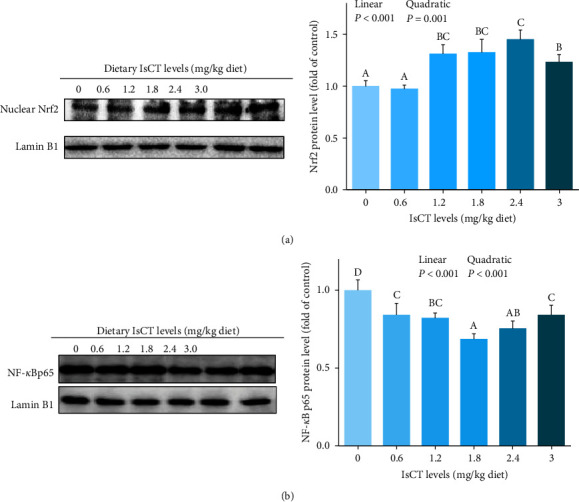
The related protein expression (a) Nrf2 and (b) NF-*κ*Bp65 in the gill of grass carp fed with IsCT for 60 days and infected with *F. columnare*. Values are means ± SD, *n* = 6 (six fish in each group). Different letters indicate significant differences (*P* < 0.05). *P* values indicate a significant linear or quadratic dose–response relationship (*P* < 0.05).

Nrf2 mRNA expression in grass carp gills showed an elevation with the rise in dietary IsCT levels. Fish that were fed with the addition of IsCT at a concentration of 1.8 mg/kg exhibited the greatest Nrf2 mRNA expression (*P* < 0.05). The *Keap1a* and *Keap1b* expression levels in gill of the fish fed dietary IsCT were dramatically downregulated (*P* < 0.05). The gill samples of the grass carp exhibited low levels of Keap1a and Keap1b mRNA expression, which were slightly elevated upon the addition of IsCT at a dosage of 1.8 mg/kg. Additionally, according to Figure 4, Nrf2 protein expression increased in the 1.2–3.0 mg/kg addition group, and there was a very significant linear or quadratic effect (*P* < 0.05).

### 3.3. The Apoptosis-Related Indicators in Grass Carp Gill

**Figure 5:**
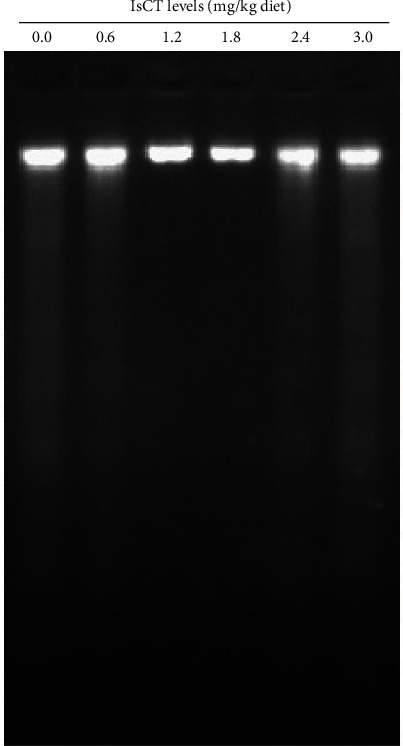
The gills of grass carp that were fed IsCT for 60 days and infected with *F. columnare* show DNA fragmentation.

According to Figure 5, the ladder-like arrangement of DNA fragments discovered in the gills of grass carp was limited by the ideal amounts of IsCT. Furthermore, the expression of *Caspase-2*, *Caspase-7*, *Apaf1*, *Fasl*, *Bax*, and *JNK* genes was significantly reduced by the addition of dietary IsCT, as shown in Figure 3. The group receiving 1.8 mg/kg IsCT supplementation exhibited the lowest expression levels (*P* < 0.05). The *Caspase-3* gene expression in grass carp fed various amounts of IsCT was notably reduced, with the lowest expression observed in the 1.2 mg/kg group (*P* < 0.05), and the expression then stabilized, showing a similar pattern for *Caspase-9*. The expression of *Caspase-8* of grass carp fed IsCT 1.8–3.0 mg/kg was concentrations were found to lower than other any groups (*P* < 0.05). Upon discontinuation of the IsCT diet at a dosage of up to 1.8 mg/kg (*P* < 0.05), there was a notable rise in the mRNA expression of *Bcl-2*, *Mcl-1*, and *IAP*, followed by a gradual decline. The expression of p38MAPK was slightly increased by the dietary intervention (*P* > 0.05).

### 3.4. The Expression of Tight Junction-Related Molecules in the Gills of Grass Carp

According to Figure 3, the expression of *JAM*, *claudin-3c*, *occludin*, *claudin-b*, *ZO-2b*, and *ZO-1* were slowly increased with the increase in the dietary IsCT level to 1.8 mg/kg (*P* < 0.05) and followed by decreases. Fish fed dietary IsCT 0.6, 1.2, and 1.8 mg/kg diets displayed relatively higher levels of *claudin-c* mRNA than those in other groups (*P* < 0.05). The expression of *claudin-11* was significantly enhanced by IsCT supplementation (*P* < 0.05), but no significant variation was observed among all IsCT supplementation groups (*P* > 0.05). The mRNA expression of *claudin-12* exhibited a gradual decrease upon reaching a dietary IsCT supplementation of 1.8 mg/kg (*P*  < 0.05), followed by a subsequent gradual increase.

### 3.5. The Main Parameters of Grass Carp Gill Immune Function

**Figure 6:**
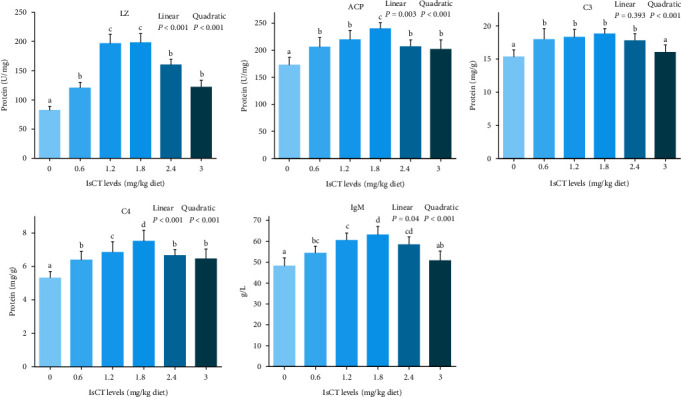
The immune parameters in the gills of grass carp fed with IsCT for a duration of 60 days and subsequently infected with *F. columnare*. Values are means ± SD, *n* = 6 (six fish in each group). Different letters indicate significant differences (*P* < 0.05). *P* values indicate a significant linear or quadratic dose–response relationship (*P* < 0.05).

The immune function parameters of grass carp gills were analyzed, and the findings were documented in Figure 6. The addition of IsCT notably increased the ACP and LZ activities, as well as the C4 and IgM levels in grass carp gills. These effects showed a significant linear or quadratic increase (*P* < 0.05). The addition of IsCT to the diet resulted in a significant quadratic increase effect on the C3 contents (*P* < 0.05). In the 1.8 mg/kg IsCT diet group, LZ, ACP activities, and the contents of C3, C4, and IgM in grass carp gill reached a peak (*P* < 0.05). However, there was no significant difference in LZ activities between the 1.8 mg/kg IsCT diet group and the 1.2 mg/kg group. Similarly, there was no significant difference in IgM content among the 1.2 and 2.4 mg/kg groups in the 1.8 mg/kg IsCT diet group (*P* > 0.05).

### 3.6. Expression of Antimicrobial Peptides and Mucin mRNA in Grass Carp Gill

**Figure 7:**
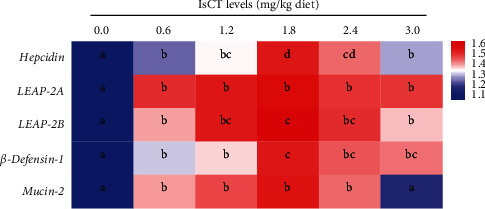
Heat-map of antimicrobial peptide-related gene expression of grass carp gill. Different letters indicate significant differences (*P* < 0.05).

The expressions of AMPs and mucins mRNA in grass carp gills were showed in Figure 7. Initially, the mRNA expression of *β-defen*sin-*1*, *hepcidin*, *mucin-2*, and *LEAP-2B* showed an upward trend and then reduced, with the highest expression observed at a dosage of 1.8 mg/kg. (*P* < 0.05). The *LEAP-2A* expression was increased with the supplementation of IsCT in the diet (*P* < 0.05), although no statistically significant differences were observed among the various IsCT supplementation groups (*P* > 0.05).

### 3.7. Expression of Cytokines Associated with Inflammation in the Gill of Grass Carp

**Figure 8:**
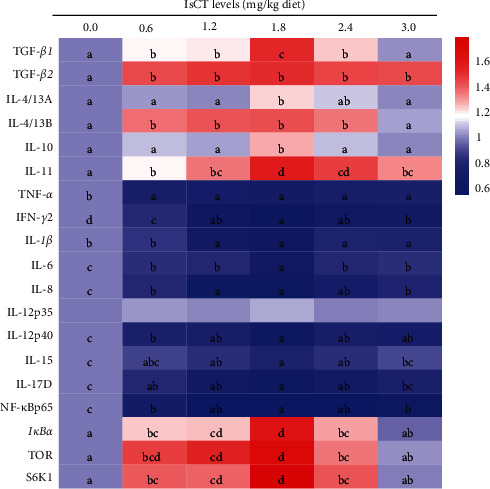
Heat-map of IsCT-changed expression of proinflammatory cytokines and anti-inflammatory cytokines. Different letters indicate significant differences (*P* < 0.05).

The expression levels of inflammation-related cytokines were documented in Figure 8. In the gills of grass carp fed the 1.8 mg/kg IsCT addition group, the expression levels of *IL-12 p40*, *IFN-γ2*, *IL-6*, *IL-1β*, *IL-8*, *IL-15*, and *IL-17D* were initially the lowest and then showed an increase (*P* < 0.05). The levels of *TNF-α* expression exhibited a significant decrease in response to the addition of IsCT to the diet at a dose of 0.6 mg/kg (*P* < 0.05), while they remained unchanged at higher doses (*P* > 0.05). Conversely, the expression of *TGF-β1*, *IL-11*, I*L-10*, and *IL-4/13A* was greatly enhanced in the group receiving a dosage of 1.8 mg/kg (*P* < 0.05). Additionally, the expression of *IL-4/13B* in the 0.6–2.4 mg/kg group surpassed that of both the 0 and 3.0 mg/kg group (*P* < 0.05). Notably, the expression of *TGF-β2* increased with the addition of IsCT to the diet at a dose of 0.6 mg/kg (*P* < 0.05) and then remained stable (*P* > 0.05).

The gill of fish that were fed diets containing IsCT at concentrations ranging from 0.6 to 2.4 mg/kg exhibited significantly elevated levels of *TOR*, *IκBα*, and *S6K1* expression compared to fish fed with alternative diets (*P* < 0.05). The group that received a supplementation of 1.8 mg/kg of IsCT showed the greatest levels of expression. Conversely, the expression of the *NF-κB p65* gene lowest in the groups supplemented with 1.2 and 1.8 mg/kg of IsCT in the diet (*P* < 0.05). Furthermore, according to Figure 4, the levels of NF-*κ*B p65 protein exhibited a decline in all the groups that received IsCT supplementation, demonstrating a notable linear or quadratic impact (*P* < 0.05).

## 4. Discussion

Recently, AMPs, as one of the most effective antibacterial drugs to control aquatic diseases, have played a crucial part in the innate immune system and protected the body from pathogenic infections [25]. The growth of grass carp fed IsCT-supplemented diets was accelerated in terms of final body weight, specific growth rate, and feed efficiency, suggesting that dietary appropriate AMPs can promote the growth of grass carp [7]. The health of fish gills is widely documented as crucial for their growth. This study provides the first analysis of alterations in gill condition in grass carp caused by the consumption of IsCT-containing diet. The primary objective of this investigation was to investigate the impact of dietary IsCT on the immune function and structural integrity of grass carp gills following infection with *F. columnare*.

### 4.1. Optimal Levels of Dietary IsCT Improved Fish Resistance to Gill Rot after *F. columnare* Infection

The proper growth of fish is closely connected to the soundness of their gills' structure [22]. *F. columnare*, a gram-negative bacterium, is the etiological agent of the columnaris disease, and it can affect the fish immune system function, oxygen homeostasis, and other processes, principally affects the epithelial tissue (including the gills) of fish [26, 27]. Gills contact with aquatic environment directly and firstly come into contact with pathogen infection [28]. Our research findings indicate that insufficient levels of IsCT led to a highest gill rot morbidity (27.13%) when infected with *F. columnare*. Markedly, grass carp in *F. columnare* groups had abnormal histological features in the gill tissue, explaining the impaired gill function, while the lowest gill rot morbidity (7.54%) in optimal IsCT supplementation (1.8 mg/kg) in grass carp. Some studies have found that AMPs can achieve antibacterial effects by destruction of the cell wall and membrane of bacteria [29]. Thus, the decrease in gill rot morbidity may be attributed to the IsCT disruption of *F. columnare* cellular structure. Accordingly, the results suggested that IsCT gives full play to its superiority effect, thus improving fish defense against the occurrence of gill damage. According to Chen et al. [30], it has been demonstrated in previous research that the consumption of AMPs APSH-07 in the diet can improve the ability of abalone *Haliotis discus hannai Ino* to resist vibriosis. There exists evidence suggesting a close association between fish gill health and the functionality of both physical and immune barriers. Consequently, we conducted additional research to explore the effects of IsCT on the physical and immune barriers of the gills.

### 4.2. IsCT Enhances the Physical Barrier Function of Grass Carp's Gill Infected with *F. columnare*

#### 4.2.1. The Antioxidant Capacity in Grass Carp Gills Was Enhanced by Consuming an Ideal Amount of IsCT in the Diet

Numerous studies have provided evidence for the detrimental effects of excessive ROS on oxidative damage and the structural integrity of gills, especially cell membranes, proteins, and lipids. MDA and PC are usually used to detect the oxidative injury of lipids and proteins in fish [31]. In the context of fish, two indices, namely, ASA and AHR, have been utilized to assess the scavenging ability of O_2^−^_• and •OH− [32]. Aside from their antibacterial and immunomodulatory effects, antimicrobial peptides also possess strong antioxidant properties [33]. Our findings proposed that the optimal IsCT level significantly decreased ROS, MDA, PC, ASA, and AHR contents in grass carp gills, implying a beneficial role of IsCT for decreasing the oxidative damage in fish gills. Antioxidant defense systems, both enzymatic and nonenzymatic, are generally considered to contribute to ROS scavenging ability. Our results showed that grass carp infected with *Flavobacterium columnare* were devoted to the overproduction of ROS in the gill, while optimal IsCT by significantly increased the GSH content, CAT, Cu/ZnSOD, GR, GPx, GST, and T-SOD activities to stimulate the gill antioxidant system and alleviate organismal oxidative damage. According to research, the addition of AMP at various levels increased the functions of T-AOC and SOD while reducing the MDA content. This could potentially enhance the antioxidant capability of the intestinal system in Pengze crucian carp (*Carassius auratus var. Pengze*) [34]. IsCT was the shortest *α*-helical AMP; the peptide bonds in AMPs can provide electrons to remove free radicals, thereby eliminating the destructive effects of free radicals on biomolecules [35]. Based on this evidence, we determined that IsCT could improve grass carp gills antioxidant capacity.

Partly, there is evidence suggesting that the levels of mRNA for antioxidant enzymes can affect their activity [36]. The present research found that the ideal IsCT in the diet increased the expression of *GPx1a*, *CuZnSOD*, *GPx1b*, *CAT*, *GPx4b*, *GSTR*, *GSTP1*, *MnSOD*, *GSTO1*, *GPx4a*, *GR*, and *GSTO2* mRNA in the gills of grass carp. Similar to Rashidian et al. [20] report, an upregulation in expression of CAT and SOD genes was observed in AMP group compared to the control in zebrafish liver that indicates the modulatory function on antioxidant capacity.

The regulation of oxidative stress is predominantly governed by Nrf2, a transcription factor responsible for modulating the expression of antioxidant enzymes in mammals. Notably, the Nrf2 signaling pathway is repressed by Keap1 [37]. In response to an ideal IsCT diet, our data showed a remarkable rise in Nrf2 mRNA expression in the gills of grass carp, along with increased levels of overall Nrf2 protein. In contrast, a reduction in the mRNA levels of *Keap1a* and *Keap1b* was noted. On the basis of these findings, we hypothesize that IsCT may modulate the expression of antioxidant enzyme mRNA in grass carp gills by upregulating the Nrf2 gene, thus improving its antioxidant capacity. It is well-known that excessive ROS diminishes antioxidant defense system, leading to cellular apoptosis. Therefore, the next step was to research the potential effects of IsCT on grass carp gill apoptosis signaling.

#### 4.2.2. The Optimal Amount of IsCT in the Diet Suppressed Cell Death in the Gills of Grass Carp

Apoptosis, an orchestrated cellular demise mechanism, serves a vital function in eradicating impaired cells caused by stress and upholding bodily equilibrium. It is commonly identified by the presence of caspase activity [38]. Apoptosis is primarily characterized by the fragmentation of DNA [39]. In this study, optimal IsCT decreased DNA fragmentation in grass carp gills. Research has indicated that the mitochondrial and the death ligand apoptotic pathways have significant involvement in mammalian apoptosis [40]. Caspase-2, caspase-3, caspase-7, and caspase-9 mediate the mitochondrial pathways. Death receptors trigger the activation of caspase-8 through Fas/FasL [41]. Our research revealed that optimal IsCT decreased the expression levels of *caspase-2*, *caspase-3*, *caspase-7*, *caspase-8*, *caspase-9*, *FasL*, *Apaf-1*, *JNK*, and *Bax* while increasing the mRNA levels of *Mcl-1*, *IAP*, *and Bcl-2* in the gill of grass carp. However, the dietary intake of IsCT did not have any impact on the mRNA levels of p38MAPK.

Antimicrobial peptides are small, multifunctional peptides synthesized in ribosomes with a relatively low molecular weight [42]. Furthermore, it is already known that low-molecular-weight substances have strong antioxidant activity [43]. Therefore, we speculate that the antiapoptotic effect of IsCT may be attributed to its strong antioxidant activity. Antimicrobial peptide JH-3 was observed to inhibit the *caspase-9* and *caspase-8* expression, further impair *caspase-3* activation, and cut down *Salmonella typhimurium* CVCC541-induced apoptosis of macrophages [44]. Our data indicated that IsCT could improve the gill physical barrier by inhibiting apoptosis.

#### 4.2.3. The Optimal Amount of IsCT in the Diet Enhanced the Integrity of Tight Junctions Partly in the Gills of Grass Carp

There is general consensus that tight junctions, such as claudins, occludin molecules, junctional adhesion molecules, and ZO-1 proteins, play a key role in the fish gill epithelium. According to Ou et al. [45], it has been stated that the breakdown of the tight junction barrier can lead to impairment of the epithelial barrier function in aquatic organisms. In our observations, dietary optimal IsCT upregulated the expression of *occludin*, *claudins* (-*b*, -*c*, -*3c*, and -*11*), *ZO-1*, and *ZO-2b* mRNA, thereby making the epithelial barrier function more complete. Many studies have demonstrated that upregulated gene expression of claudin-12 implies the disrupted intercellular integrity [11]. The gene expression of *claudin-12* was reduced in our findings. Amphipathic and *α*-helical structure in AMPs have the capacity to modulate epithelial barrier function [46], potentially explaining the observed improvement in the tight junction barrier function of the gill with IsCT supplementation.

In previous studies, it has been shown that the phosphorylation of myosin II regulatory light chain (MLC) by myosin light chain kinase (MLCK) plays a role in signal transduction pathways and subsequently controls the regulation of the tight junction barrier in response to certain external stimuli [47]. In our research, we discovered that including IsCT in the diet led to a decrease in the expression of *MLCK* mRNA in the gill of grass carp. It is important to mention that our prior investigation into the influence of IsCT levels on the gastrointestinal tract produced a comparable result [33]. The results of this study suggest that IsCT may enhance the expression of *TJs* mRNA in grass carp gills, potentially leading to a decrease in *MLCK* mRNA levels.

### 4.3. IsCT Enhances the Immune Responses of Grass Carp's Gill Infected with *F. columnare*

#### 4.3.1. Appropriate Level of IsCT Improvement of Antibacterial Ability in Grass Carp Gills

The AMPs are the natural components of the innate immune system, and apart from their antimicrobial activity, they also play a central role in guarding living organisms against microbial invasions in fish [20]. AMPs are secreted by the mucus layer of fish gills as a first-line barrier against pathogenic bacteria [48]. Lysozyme (LZ) is a humoral component with bactericidal activity in the fish innate immune system [49, 50]. Our results revealed that appropriate dosages of IsCT augment the LZ activity in grass carp gills. The results of studies on LZ activity in the serum of *Asian catfish Clarias batrachu* were similar [51]. Acid phosphatase (ACP) is an important component of lysosomes, which play a role in the immune response to kill and digest pathogens [30]. In our present experiment, the activities of ACP increased at first and then decreased with increasing dietary IsCT. In addition to dissolving foreign cells, fish complement components can destroy phagocytes, which regulate foreign organisms [52]. Immunoglobulin M (IgM), mucin-2, and hepcidin were essential for fish to combat bacterial and viral infections [53, 54]. In particular, our research shows that the concentrations of C3, C4, and IgM initially rose and subsequently declined when exposed to higher IsCT levels in the diet. In the meantime, the levels of *LEAP-2A*, *hepcidin*, *LEAP-2B*, *β-defen*sin-*1*, and *mucin-2* showed an increase upon IsCT feeding, suggesting that IsCT enhances the immune function in the gill of grass carp.

Our findings were in line with previous studies who reported that supply of AMPs could improve the content of ACP, LZ, and IgM in common carp serum [55]. Therefore, we infer that the optimal amount of IsCT could enhance the innate immune response of grass carp gill. A dominant role played by AMPs is in killing bacteria, but they may also be able to suppress inflammation as well. The relationship between immunity and inflammation response was closely related [56]. Consequently, we will analyze the impact of IsCT on the inflammation of grass carp's gills.

#### 4.3.2. Appropriate Level of IsCT Partially Reduce Inflammatory Response by NF-*κ*Bp65 and TOR Signaling Pathways in Grass Carp Gills

According to previous studies, there have been published that the involvement of cytokines in inflammation plays a crucial role during the initial phase of the cellular immune response in fish [57]. Generally, most studies showed the beneficial effects of AMPs in terms of improving the immune system [58]. Up to now, no study has been estimated the effects of AMPs as feed additives in fish gills, so it is difficult to make a direct comparison. Our findings indicate that the intake of a diet rich in IsCT significantly suppressed the mRNA expression of proinflammatory cytokines (*IL-1β*, *IL-17D*, *IL-8*, *IFN-γ2*, *IL-12 p40*, *TNF-α*, *IL-15*, and *IL-6*) while simultaneously enhancing the mRNA expression of anti-inflammatory cytokines (*IL-10*, *TGF-β1*, *TGF-β2*, *IL-4/13A*, *IL-4/13B*, and *IL-11*). These persistent anti-inflammatory signals and the reduced state of inflammation suggest that IsCT has the potential to alleviate inflammation in the gills of grass carp. This is consistent with previous findings that AMPs induced the production of *TNF-α* and *IL-1β* mRNA in both the head kidney and trunk kidney of *Epinephelus coioides* [25]. Prior research have reveal that administering the antimicrobial peptide led to a decrease in the levels of *IL-6* and *IL-8* in RAW 264.8 cells stimulated with LPS [59]. Evidence shows that AMPs like Cecropin AD can boost turbot (*Scophthalmus maximus L*.) immunity and reduce mortality challenging with *Edwardsiella tarda* [60]. AMPs can induce the secretion of cytokines, thereby recruiting macrophages to play an immunomodulatory role and improving the body's resistance to pathogenic microorganisms [20]. In fish, the expression of inflammatory cytokine genes is crucially aided by the NF-*κ*B and TOR signaling pathways, which generally regulate cytokine production. So, we further elucidate the potential mechanisms of IsCT in the gill inflammatory response.

It has been found that NF-*κ*Bp65 is one of the subunits of NF-*κ*B in zebrafish [61]. The phosphorylation of I*κ*B proteins in fish is directly linked to the nuclear translocation of NF-*κ*B, which is widely recognized. Based on current findings, we observed that IsCT downregulated the *NF-κBp65* mRNA expression and upregulated the expression of *IκBα*. AMPs CC34, harmonize with the present findings, have the ability to inhibit the NF-*κ*B signaling pathway, controlling the mRNA levels of TNF-*α*, IL-1*β*, and IL-6 genes and reducing inflammation in the intestines of mice [62]. A study found that the antimicrobial peptide YD1 has anti-inflammatory properties by inhibiting TNF-*α* and IL-6 through the NF-*κ*B pathway and increasing HO-1 expression in RAW 264.7 cells [63]. This suggests that IsCT may help control inflammation by affecting the I*κ*B*α*/NF-*κ*Bp65 pathway.

Furthermore, the TOR signaling pathway and the phosphorylated key effectors of S6K1 could modulate inflammatory cytokine production in fish [64]. This study represents the first assessment the impact of IsCT on the TOR signaling pathway in the gills of grass carp. The present research is the initial exploration of the influence of IsCT on the TOR signaling pathway in the gills of grass carp. Our results illustrated that IsCT increased transcription of *TOR* and *S6K1* mRNA. The findings from our study suggest that the consumption of IsCT in the diet may have the ability to modulate the expression of genes related to inflammation through the TOR signaling pathway in grass carp. There is, however, a need for further research. Additionally, it is the initial comprehensive molecular examination of the effects of IsCT on gene expression related to physical barrier and immune function in grass carp gills following infection with *F. columnare*.

Thus, optimal IsCT significantly enhances the immune functions in the gills of grass carp by modulating the NF-*κ*B canonical signaling pathway mediated by I*κ*Ba/NF-*κ*Bp65 and the TOR/S6K1 signaling pathway.

## 5. Conclusions

**Figure 9:**
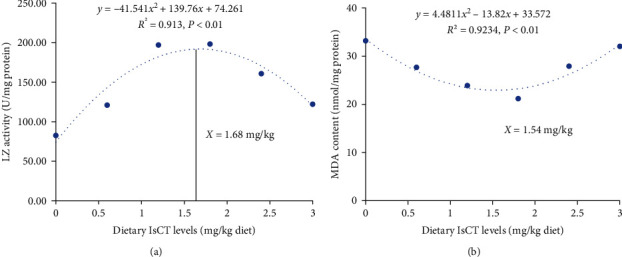
Quadratic regression analysis of LZ activities (a) and MDA content (b) for grass carp fed diets containing various IsCT levels for 10 weeks. LZ, Lysozyme; MDA, malondialdehyde.

Based on the above-mentioned studies, an appropriate level of IsCT has possible advantages in enhancing grass carp gill disease resistance, the physical barrier, and the immune function after infection with *F. columnare*. The specific results are as follows: (1) dietary IsCT demonstrates a potential for mitigating oxidative damage, relieving cell apoptosis, and enhancing the tight junction barrier to improve grass carp gill physical barrier function. Its promoting function seems to act by modulating the Nrf2, JNK, and MLCK signaling pathway, respectively. (2) The immune barrier function of grass carp gills was enhanced by the consumption of IsCT in their diet. A combination of anti-inflammatory cytokines and antimicrobial compounds suppressed inflammation by increasing levels of anti-inflammatory cytokines and reducing proinflammatory cytokines. The optimal dietary IsCT was found to regulate the TOR and NF-*κ*B p65 signaling pathways, leading to changes in immune function. In addition, based on LZ activity and against lipid peroxidation, optimum IsCT concentrations in on-growing grass carp (136.88 ± 0.72 g) were found to be 1.68 and 1.54 mg/kg diet (Figure 9), respectively.

## Data Availability

The data behind this article will be shared with the appropriate authors upon reasonable request.

## Supplementary Materials

Supplementary Materials The original contributions presented in the study are included in the article/Supplementary Material. Further inquiries can be directed to the corresponding author.
