# Supplementary material for: Exploring the Potential of Isalo Scorpion Cytotoxic Peptide in Enhancing Gill Barrier Function and Immunity in Grass Carp (Ctenopharyngodon idella) Infected with Flavobacterium columnare
Source: Aquac Nutr. 2024 Jul 24;2024:8059770. doi: 10.1155/2024/8059770 (PMC11300071; doi:10.1155/2024/8059770)
Supplement: Supplementary Materials — The original contributions presented in the study are included in the article/Supplementary Material. Further inquiries can be directed to the corresponding author. [file 8059770.f1.docx]

**Abbreviations**

**AHR**, Anti-hydroxyl radical; **ASA**, Anti-superoxide anion; **Bcl-2**, B-cell lymphoma-2; **Bax**, B-cell lymphoma protein 2 associated X protein; **ACP**, Acid phosphatase; **AMPs**, Antimicrobial peptides; **CAT**, Catalase; **CuZnSOD**, copper/zinc superoxide dismutase; **Caspase**, Cysteinyl aspartate specific proteinase; **C3**, Complement 3; **C4**, Complement 4; **FasL**, Fas ligand; **GSH**, Glutathione; **GSH-Px**, Glutathione peroxidase; **GR**, Glutathione reductase; **IgM**, Immunoglobulin M; **IL**, Interleukin; **IFN-γ2**, Interferon γ2; **IκBα**, Inhibitor of κBα; **IsCT**, Isalo scorpion cytotoxic peptide; **Keap1**, Kelch-like ECH-associated protein 1; **LZ**, Lysozyme activity; **MDA**, Malondialdehyde; **Mn-SOD**, Manganese superoxide dismutase; **Nrf2**, Nuclear factor-erythroid 2-related factor 2; **Mcl-1**, Myeloid cell leukemia-1; **NF-κB**, Nuclear factor κB; **•OH**, Hydroxyl radicals; **O2•^−^**, Superoxide anions; **PC**, Protein carbonyl; **ROS**, Reactive oxygen species; **TJs**, Tight junctions; **TGF-β**, Transforming growth factor β; **TNF-α**, Tumor necrosis factor α; **TOR**, Target of rapamycin; **ZO-1**, Zonula occluden-1.

**Table S1** Formulation and nutrient content of the basal diet

| Ingredients | Content (%) | Nutrients content | Content (%) |
| --- | --- | --- | --- |
| Fish meal | 5.00 | Crude protein ^5^ | 28.60 |
| Soybean meal | 20.90 | Crude lipid | 5.02 |
| Cottonseed meal | 11.70 | n3 ^6^ | 1.04 |
| Rapeseed meal | 14.07 | n6 ^6^ | 0.96 |
| Rice gluten meal | 8.00 | Available phosphorus ^7^ | 0.40 |
| α-Starch | 28.00 |  |  |
| Corn starch | 2.08 |  |  |
| Fish oil | 2.67 |  |  |
| Soybean oil | 1.06 |  |  |
| Ca(H_2_PO_4_)_2_ | 1.38 |  |  |
| Vitamin premix ^1^ | 1.00 |  |  |
| Mineral premix ^2^ | 2.00 |  |  |
| Choline chloride (50%) ^3^ | 1.00 |  |  |
| Ethoxyquin (30%) | 0.05 |  |  |
| AMP premix ^4^ | 1.00 |  |  |
| Thr (98.5%) | 0.09 |  |  |

^1^ Vitamin premix (g/kg): retinyl acetate (1,000,000 IU/g), 0.19; cholecalciferol (500,000 IU/g), 0.20; L-a-tocopherol acetate (50%), 23.23; menadione(96%), 1.98; cyanocobalamin (1%), 0.94; D-biotin (2%), 0.75; folic acid (95%),0.17; thiamine nitrate (98%), 0.09; ascorhyl acetate (95%), 9.77; niacin (99%),3.44; meso-inositol (97%), 28.53; calcium-D-pantothenate (90%), 4.19; riboflavin (80%), 0.73; pyridoxine hydrochloride (98%), 0.45; All ingredients were diluted with corn starch to 1 kg.

^2^ Per kilogram of mineral premix (g/kg): MnSO_4_. H_2_O (31.8% Mn), 2.6590; MgSO_4_.H2O (15.0% Mg), 256.7933; FeSO_4_. H_2_O (30.0% Fe), 12.6083; ZnSO_4_. H_2_O (34.5% Zn), 8.8700; CuSO_4_. 5H_2_O (25.0% Cu), 0.9560; CaI (3.2% I), 1.5625; Na_2_SeO_3_ (44.7% Se), 0.0611; All ingredients were diluted with corn starch to 1kg.

^3^ Choline chloride premix (g/kg premix): Choline chloride premix (50%), 261.90 g; All ingredients were diluted with corn starch to 1 kg.

^4^ AMP premix: premix was added to obtain graded levels of AMP, and the amount of maize starch was reduced to compensate.

^5^ Crude protein and crude lipid contents were measured values.

^6^ n-3 and n-6 contents were referenced to Zeng et al., and calculated according to NRC (2011) (Resources, 2011; Zeng et al., 2016).

^7^ Available phosphorus was referenced to Wen et al. (2015) and calculated according to NRC et al. (2011) (Resources, 2011; Wen et al., 2015).

**Table S2** Real-time PCR sequences.

| Genes | Primer sequence Forward (5^’^→3^’^) | Primer sequence Reverse (5^’^→3^’^) | Accession number |
| --- | --- | --- | --- |
| Cu/ZnSOD | CGCACTTCAACCCTTACA | ACTTTCCTCATTGCCTCC | GU901214 |
| MnSOD | ACGACCCAAGTCTCCCTA | ACCCTGTGGTTCTCCTCC | GU218534 |
| CAT | GAAGTTCTACACCGATGAGG | CCAGAAATCCCAAACCAT | FJ560431 |
| GPx1a | GGGCTGGTTATTCTGGGC | AGGCGATGTCATTCCTGTTC | EU828796 |
| GPx1b | TTTTGTCCTTGAAGTATGTCCGTC | GGGTCGTTCATAAAGGGCATT | KT757315 |
| GPx4a | TACGCTGAGAGAGGTTTACACAT | CTTTTCCATTGGGTTGTTCC | KU255598 |
| GPx4b | CTGGAGAAATACAGGGGTTACG | CTCCTGCTTTCCGAACTGGT | KU255599 |
| GSTR | TCTCAAGGAACCCGTCTG | CCAAGTATCCGTCCCACA | EU107283 |
| GSTP1 | ACAGTTGCCCAAGTTCCAG | CCTCACAGTCGTTTTTTCCA | KM112099 |
| GSTP2 | TGCCTTGAAGATTATGCTGG | GCTGGCTTTTATTTCACCCT | KP125490 |
| GSTO1 | GGTGCTCAATGCCAAGGGAA | CTCAAACGGGTCGGATGGAA | KT757314 |
| GSTO2 | CTGCTCCCATCAGACCCATTT | TCTCCCCTTTTCTTGCCCATA | KU245630 |
| GR | GTGTCCAACTTCTCCTGTG | ACTCTGGGGTCCAAAACG | JX854448 |
| Nrf2 | CTGGACGAGGAGACTGGA | ATCTGTGGTAGGTGGAAC | KF733814 |
| keap1a | TTCCACGCCCTCCTCAA | TGTACCCTCCCGCTATG | KF811013 |
| keap1b | TCTGCTGTATGCGGTGGGC | CTCCTCCATTCATCTTTCTCG | KJ729125 |
| caspase-2 | CGCTGTTGTGTGTTTACTGTCTCA | ACGCCATTATCCATCTCCTCTC | KT757313 |
| caspase-3 | GCTGTGCTTCATTTGTTTG | TCTGAGATGTTATGGCTGTC | JQ793789 |
| caspase-7 | GCCATTACAGGATTGTTTCACC | CCTTATCTGTGCCATTGCGT | KT625601 |
| caspase-8 | ATCTGGTTGAAATCCGTGAA | TCCATCTGATGCCCATACAC | KM016991 |
| caspase-9 | CTGTGGCGGAGGTGAGAA | GTGCTGGAGGACATGGGAAT | JQ793787 |
| Apaf-1 | AAGTTCTGGAGCCTGGACAC | AACTCAAGACCCCACAGCAC | KM279717 |
| Bax | CATCTATGAGCGGGTTCGTC | TTTATGGCTGGGGTCACACA | JQ793788.1 |
| Bcl-2 | AGGAAAATGGAGGTTGGGAT | CTGAGCAAAAAAGGCGATG | JQ713862.1 |
| Mcl-1 | TGGAAAGTCTCGTGGTAAAGCA | ATCGCTGAAGATTTCTGTTGCC | KT757307 |
| IAP | CACAATCCTGGTATGCGTCG | GGGTAATGCCTCTGGTGCTC | FJ593503.1 |
| FasL | AGGAAATGCCCGCACAAATG | AACCGCTTTCATTGACCTGGAG | KT445873 |
| JNK | ACAGCGTAGATGTGGGTGATT | GCTCAAGGTTGTGGTCATACG | KT757312 |
| p38MAPK | TGGGAGCAGACCTCAACAAT | TACCATCGGGTGGCAACATA | KM112098 |
| ZO-1 | CGGTGTCTTCGTAGTCGG | CAGTTGGTTTGGGTTTCAG | KJ000055 |
| ZO-2b | TACAGCGGGACTCTAAAATGG | TCACACGGTCGTTCTCAAAG | KM112095 |
| occludin | TATCTGTATCACTACTGCGTCG | CATTCACCCAATCCTCCA | KF193855 |
| claudin-b | GAGGGAATCTGGATGAGC | ATGGCAATGATGGTGAGA | KF193860 |
| claudin-c | GAGGGAATCTGGATGAGC | CTGTTATGAAAGCGGCAC | KF193859 |
| claudin-3c | ATCACTCGGGACTTCTA | CAGCAAACCCAATGTAG | KF193858 |
| claudin-11 | TTTCTGGTTCACTTCCGAGG | TCTCAACTGCTCTGTATCACTGC | KT445867 |
| claudin-12 | CCCTGAAGTGCCCACAA | GCGTATGTCACGGGAGAA | KF998571 |
| MLCK | GAAGGTCAGGGCATCTCA | GGGTCGGGCTTATCTACT | KM279719 |
| JAM | ACTGTGAGGTGCTTGGAA | CTGTTGTGACTGAAGAAGGA | KY780630 |
| hepcidin | AGCAGGAGCAGGATGAGC | GCCAGGGGATTTGTTTGT | JQ246442.1 |
| LEAP-2A | TGCCTACTGCCAGAACCA | AATCGGTTGGCTGTAGGA | FJ390414 |
| LEAP-2B | TGTGCCATTAGCGACTTCTGAG | ATGATTCGCCACAAAGGGG | KT625603 |
| β-defensin | TTGCTTGTCCTTGCCGTCT | AATCCTTTGCCACAGCCTAA | KT445868 |
| Mucin-2 | GAGTTCCCAACCCAACACAT | AAAGGTCTACACAATCTGCCC | KT625602 |
| TNF-α | CGCTGCTGTCTGCTTCAC | CCTGGTCCTGGTTCACTC | HQ696609 |
| IFN-γ2 | TGTTTGATGACTTTGGGATG | TCAGGACCCGCAGGAAGAC | JX657682 |
| IL-1β | AGAGTTTGGTGAAGAAGAGG | TTATTGTGGTTACGCTGGA | JQ692172 |
| IL-6 | CAGCAGAATGGGGGAGTTATC | CTCGCAGAGTCTTGACATCCTT | KC535507.1 |
| IL-8 | ATGAGTCTTAGAGGTCTGGGT | ACAGTGAGGGCTAGGAGGG | JN663841 |
| IL-12p35 | TGGAAAAGGAGGGGAAGATG | AGACGGACGCTGTGTGAGTGTA | KF944667.1 |
| IL-12p40 | ACAAAGATGAAAAACTGGAGGC | GTGTGTGGTTTAGGTAGGAGCC | KF944668.1 |
| IL-15 | CCTTCCAACAATCTCGCTTC | AACACATCTTCCAGTTCTCCTT | KT445872 |
| IL-17D | GTGTCCAGGAGAGCACCAAG | GCGAGAGGCTGAGGAAGTTT | KF245426.1 |
| TGF-β1 | TTGGGACTTGTGCTCTAT | AGTTCTGCTGGGATGTTT | EU099588 |
| TGF-β2 | TACATTGACAGCAAGGTGGTG | TCTTGTTGGGGATGATGTAGTT | KM279716 |
| IL-4/13A | CTACTGCTCGCTTTCGCTGT | CCCAGTTTTCAGTTCTCTCAGG | KT445871 |
| IL-4/13B | TGTGAACCAGACCCTACATAACC | TTCAGGACCTTTGCTGCTTG | KT625600 |
| IL-10 | AATCCCTTTGATTTTGCC | GTGCCTTATCCTACAGTATGTG | HQ388294 |
| IL-11 | GGTTCAAGTCTCTTCCAGCGAT | TGCGTGTTATTTTGTTCAGCCA | KT445870 |
| NF-κB p65 | GAAGAAGGATGTGGGAGATG | TGTTGTCGTAGATGGGCTGAG | KJ526214 |
| IκBα | TCTTGCCATTATTCACGAGG | TGTTACCACAGTCATCCACCA | KJ125069 |
| TOR | TCCCACTTTCCACCAACT | ACACCTCCACCTTCTCCA | JX854449 |
| S6K1 | TGGAGGAGGTAATGGACG | ACATAAAGCAGCCTGACG | EF373673 |
| β-actin | GGCTGTGCTGTCCCTGTA | GGGCATAACCCTCGTAGAT | M25013 |

CuZnSOD, copper, zinc superoxide dismutase; MnSOD, manganese superoxide dismutase; CAT, catalase; GPx, glutathione peroxidase; GST, glutathione-Stransferase; GR, glutathione reductase; Nrf2, Nuclear factor-erythroid 2-related factor 2; Bax, B-cell lymphoma protein 2 associated X protein; Bcl-2, B-cell lymphoma-2; Mcl-1, Myeloid cell leukemia-1; FasL, Fas ligand; Apaf-1, apoptotic protease-activating factor 1; JNK, c-Jun N-terminal kinase;JAK, janus kinase; IAP, inhibitor of apoptosis; ZO-1, zonula occludens-1; MLCK, myosin light chain kinase; JAM, junctional adhesion molecule; NMII, non-muscle myosin II; IL, Interleukin; IFN-γ2, Interferon γ2; IκBα, Inhibitor of κBα; TGF-β, Transforming growth factor β; TNF-α, Tumor necrosis factor α; TOR, Target of rapamycin; NF-κB, Nuclear factor κB; S6K1, protein S6 kinase 1.
